# Hypoglycemia event prediction from CGM using ensemble learning

**DOI:** 10.3389/fcdhc.2022.1066744

**Published:** 2022-12-09

**Authors:** Jesper Fleischer, Troels Krarup Hansen, Simon Lebech Cichosz

**Affiliations:** ^1^ Steno Diabetes Center Aarhus, Aarhus, Denmark; ^2^ Steno Diabetes Center Zealand, Holbæk, Denmark; ^3^ Department of Health Science and Technology, Aalborg University, Aalborg, Denmark

**Keywords:** diabetes, machine learning, hypoglycaemia, type 1 diabetes, continuous glucose monitoring (CGM), event prediction, Dexcom G4 platinum, blood glucose (BG)

## Abstract

This work sought to explore the potential of using standalone continuous glucose monitor (CGM) data for the prediction of hypoglycemia utilizing a large cohort of type 1 diabetes patients during free-living. We trained and tested an algorithm for the prediction of hypoglycemia within 40 minutes on 3.7 million CGM measurements from 225 patients using ensemble learning. The algorithm was also validated using 11.5 million synthetic CGM data. The results yielded a receiver operating characteristic area under the curve (ROC AUC) of 0.988 and a precision-recall area under the curve (PR AUC) of 0.767. In an event-based analysis for predicting hypoglycemic events, the algorithm had a sensitivity of 90%, a lead-time of 17.5 minutes and a false-positive rate of 38%. In conclusion, this work demonstrates the potential of using ensemble learning to predict hypoglycemia, using only CGM data. This could help alarm patients of a future hypoglycemic event so countermeasures can be initiated.

## Introduction

Hypoglycemia is related to both increased physical, and mental health problems and is a major risk factor for mortality (1, 2). Hypoglycemia can result from exogenous or endogenous insulin excess alone. The clinical manifestation is often characteristic, but the neurogenic and neuroglycopenic symptoms of hypoglycemia are nonspecific and relatively insensitive (3). Consequently, many episodes of hypoglycemia are not recognized or treated late in the progression (3). It is very important to prevent, identify and treat hypoglycemic events secondary to the use of insulin. Additionally, it is safer for the patients and more effective to prevent hypoglycemia than to treat it after it occurs (4).

Hypoglycemia is common among patients with insulin dependent diabetes. Patients who aim for a strict glycemic target experience frequent episodes of asymptomatic hypoglycemia and severe hypoglycemia (5). Studies suggest that plasma glucose levels may be less than 60 mg/dL (3.3 mmol/L) up to 10% of the day (6, 7). Furthermore, patients with type 1 diabetes suffer from an average of two weekly incidents of symptomatic hypoglycemia (6, 7).

However, newer studies on patients utilizing continuous glucose monitoring (CGM) have shown that time below range (< 3.9 mmol/L) was estimated to be 5.4% with a mean HbA1c of 7.0% (52 mmol/mol) (8).

Blood glucose prediction is about forecasting a patient’s future blood glucose levels using current and past information and is also an important constituent of blood glucose anomaly classification approaches. One potential method to reduce episodes of hypoglycemia is prediction models that can alarm patients early to begin countermeasures. Such models can be implemented directly into the CGM systems or as an add-on in the patient’s smartphone applications connected to the systems (9).

We have in previous studies (10–13) investigated the potential of using a continuous glucose monitor (CGM) combined with heart rate variability (HRV) to predict hypoglycemia for the purpose of early intervention. Also, many others have reported the potential of predicting future glucose levels using CGM combined with multiple data sources such as insulin, physical activity, food intake, and stress response (14, 15). Obtaining these multiple data in real time is not always practical (9). Also, most studies that utilize only CGM data as a more practical approach, are often based on limited number of patients, short CGM wear-time, and are not validated in external cohorts of patients (9, 14, 16). Therefore, we sought to further explore the potential of using only CGM data for the prediction of hypoglycemia in a proof of concept analysis using a large cohort of type 1 diabetes patients during normal daily living and validating the results in an external CGM database.

## Methods

The study cohort comprised CGM data derived from individuals who were enrolled in the REPLACE-BG trial (17). The REPLACE-BG study design was a 6-month parallel group multicenter randomized clinical trial. A total of 225 patients ≥18 years of age (mean ± standard deviation or median (interquartile range): age: 44 ± 14 years, duration of diabetes: 23 ± 12 years, BMI: 27.7 ± 4.1, HbA1c: 7.1 ± 0.7% (54 mmol/mol), time in range: 63 ± 13%, time below <70 mg/dL: 2.9% (1.5–5.1)) with type 1 diabetes were enrolled from the diabetes clinics and used CGM (Dexcom G4) for up to 6 months. The characteristics are presented in **Table 1**.

**Table 1:** patients characteristics presented as mean ± standard deviation for parametric characteristics or Median (interquartile range) for non-parametric.

Age (years)	44 ± 14
- range	19-78
Diabetes duration (years)	23 ± 12
- range	2-64
BMI (kg/m2)	27.7 ± 4.1
HbA1c (%)	7.1 ± 0.7
Female sex (%)	50
Time in range (%)	63 ± 13
Time below <70 mg/dL (%)	2.9 (1.5–5.1)
Mean glucose (mg/dL)	162 ± 22

We trained and tested an algorithm for the prediction of hypoglycemia within 40 minutes on 3.7 million CGM measurements from 225 patients using an ensemble learning approach named RUSBoost (18). In short, ensemble learning is a general meta-approach to machine learning that seeks better performance by combining the predictions from multiple models. RUSBoost has been reported to be a fast and robust classifier for datasets with imbalanced data. For training, 70% of the data were utilized (split on a patient level) and the remaining 30% were reserved for testing the performance of the final model.

The hyperparameter estimation (learning cycles, learn rate, max splits) were determined using 5-fold cross-validation on the training data using a grid search strategy. A hyperparameter is a parameter whose value is used to control the learning process of the prediction model. Grid search is a specific tuning strategy that attempts to compute the optimum values of the hyperparameters. It is an exhaustive search that is performed on the specific parameter values of a model. Cross-validation is used in the process to ensure that the model is not over-tuned, which could result in worse performance on new patient data.

Input to the model was CGM data one hour prior to the point of prediction. Hypoglycemia was defined as CGM values below 70 mg/dL for 15 minutes or more (sustained hypoglycemia) – the definition was based on the recommendations in previous studies (19, 20). The algorithm was implemented using MATLAB R2020b (The Mathworks Inc., Natick, Massachusetts).

In addition to the data from real patients the algorithm was also tested on 11.5 million synthetic CGM data from the publicly available SCGMS database (18). The database mimics CGM data from type 1 patients and healthy individuals with different HbA1c levels using a Conditional Generative Adversarial Network (CGAN) (21). In short, CGAN is a novel method to construct a neural network which can be used to generate realistic biological signals. The external validation was conducted to determine the generalizability of the model in people with different glycemic control.

To evaluate the performance of the trained model we conducted a sample-based assessment that comprised every datapoint in the test dataset (real patients) and synthetic dataset. The sample-based performance was assessed using Receiver operating characteristic (ROC) and precision-recall curve with (PR curve) with accompanying area under the curve (AUC). The metrics from the sample-based assessment are important for between model comparison. However, from a clinical or patient perspective, an event-based assessment is more useful for evaluating the performance.

Therefore, we conducted an event-based assessment that was conducted on each episode of hypoglycemia to test how many episodes of hypoglycemia was detected, the lead-time (prediction time) and the number of false positives. The event-based assessment was included to assess the performance on clinical in-use situations.

## Results

### Sample-based test results

**Figure 1:**
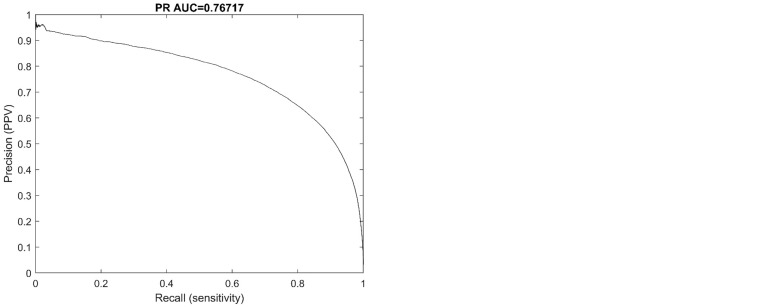
ROC and PR curves of the sample-based performance from the test dataset (real patients).

From 1,110,000 samples in the test dataset the performance of the algorithm was a receiver operating characteristic areal under the curve (ROC AUC) of 0.988 and a precision-recall area under the curve (PR AUC) of 0.767. The ROC and PR curves are illustrated in **Figure 1**.

From the 11,500,000 samples of synthetic data the assessment yielded an operating characteristic areal under the curve (ROC AUC) of 0.988 and a precision-recall area under the curve (PR AUC) of 0.879.

### Event-based test results

The results from the event-based assessment yielded a sensitivity of 90%, a lead-time of 17.5 minutes and a false-positive rate of 38%. Due to the class imbalance (few events compared to non-events) the specificity and negative-predictive-value are both high >99%. The prediction was on average triggered with glucose levels of 83 mg/dL.

Translated to round estimates this would mean that 9 out of 10 hypoglycemia events were detected on average 17 minutes prior to the first CGM value below 70 mg/dL and with 2/3 alarms being true. The metrics are calculated from a total of 3725 hypoglycemic events in the test dataset. The test dataset comprised of 5,456,905 minutes of CGM wear time during daily living.

**Figure 2:**
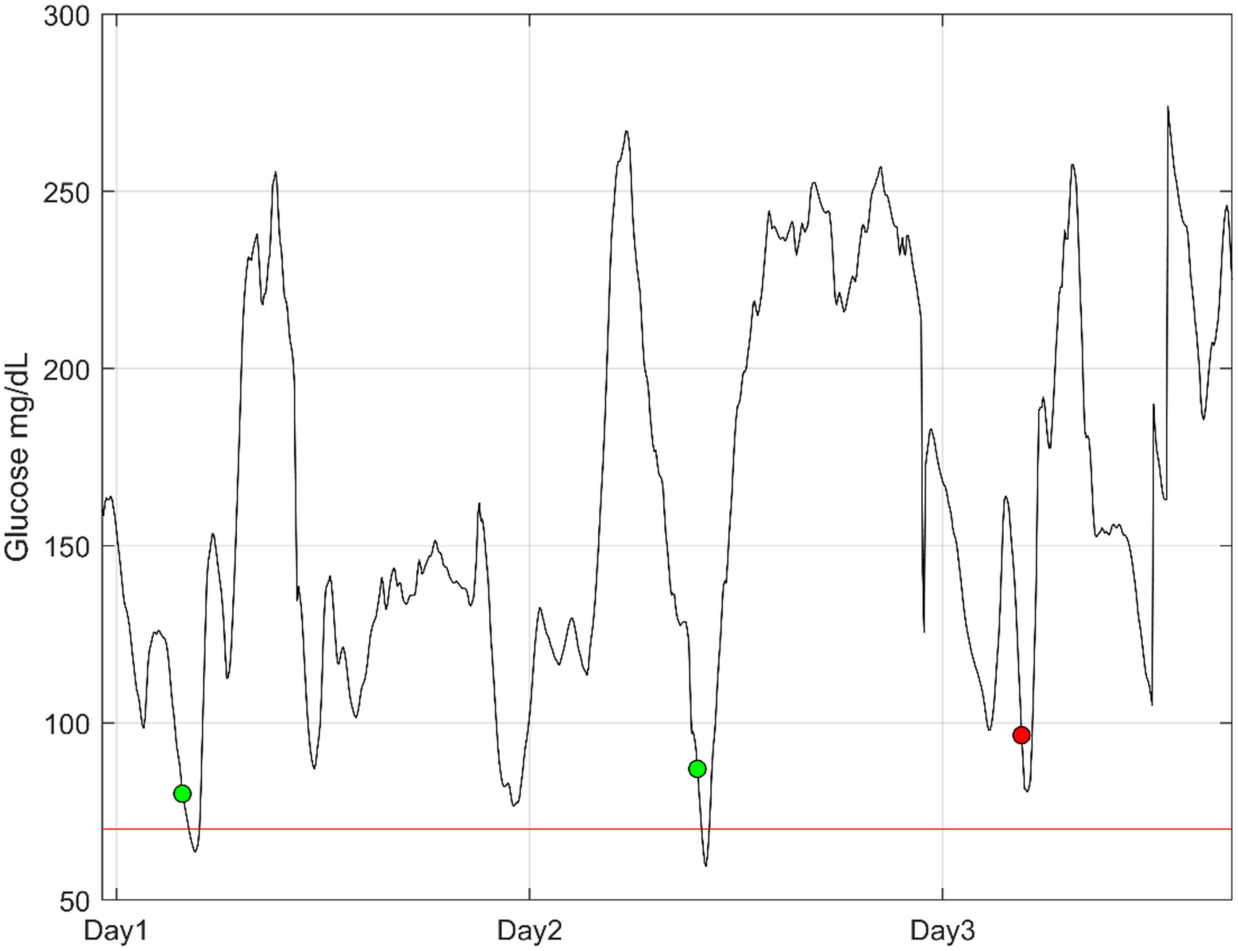
An example of three days of continuous glucose monitoring from a patient. The dots illustrate the point in time where an alarm is activated for high hypoglycemic risk. The green dots are true positives, and the red dot is a false alarm. The red line is the threshold for hypoglycemia (70 mg/dL).

**Figure 2** shows an example of prediction from three days of CGM wear. The patient would be alarmed three times, where the first two alarms are true positives, while the last is a rapid decline in glucose levels that does not lead to hypoglycemia.

## Discussion

The event-based assessment shows that it is possible to predict a large proportion of hypoglycemic events with a lead-time which makes it possible for the patients to reverse the situation and potentially avoid severe hypoglycemia. Especially during nights and rapid dips in glucose it is extremely important to be aware of the risk and start timely treatment with ingestion of fast absorbable carbohydrates or potentially glucagon to avoid severe hypoglycemia related complications. Early prediction of hypoglycemia and herby earlier intervention could also potentially aid in reducing the time in hypoglycemia.

In comparison, the commercial system Dexcom 6G advertise hypoglycemia prediction up to 20 minutes prior to hypoglycemia defined at a lower threshold of 55 mg/dL (18). However, without data on the average lead-time, sensitivity and false-positive it is difficult to compare the predictive capabilities. However, improving on the prediction capability as in our study with event prediction up to 40 minutes a-head (average lead-time 17.5 minutes) prior to an event (<70 mg/dL) would enable faster action to avoid mild hypoglycemia/sever hypoglycemia. Accurate prediction models could also be used in a closed-loop system to suspend insulin dosing in order to avoid severe hypoglycemia.

Recent studies by Darpit et al. (15, 22) have reported interesting results on the multisource prediction of hypoglycemia using a battery of features from CGM, insulin, meal intake and demographic data. They reported from a cohort of 110 pediatric patients an accuracy of predicted events with >97% sensitivity and specificity and false alert rate <25%. However, due to the difference in sensor model, methodically assessment and cohort characteristics between the studies it is challenging to compare results head-to-head and conclude if the use of additional data is worth the practical implications. CGM based hypoglycemic event prediction seems like an attractive approach due to the simplicity of implementing it into already running commercial CGM sensors or analytic platforms for CGM data.

Additionally, Seo et al. (23) proposed a model for predicting postprandial hypoglycemia using CGM and meal announcement. The study explored retrospective CGM datasets of 104 people who had experienced at least one hypoglycemia event during a three-day CGM session. The best performance reported in the study was an average AUC of 0.966, average sensitivity of 89.6%, and average specificity of 91.3%. Marcus et al. (24) published results from 11 patients with type 1 diabetes - they proposed a prediction model for hypoglycemia with a sensitivity of 64% and a low false-positive rate of 4%.

This study has some limitations; the proposed model in this study, still needs to be tested in a broader spectrum of patients and CGM sensors. One limitation in this study is that we cannot generalize the performance to all CGM sensors. Many new sensors are emerging from different manufacturers with better accuracy and decision support, such as trend arrows.

Alarm fatigue is a relevant challenge, which is why false alarm needs to be low. In our study, the proposed model, if implemented, would result in one alarm each ~10 days of wear time. This is dependent on the population and degree of glycemic control, so we cannot extrapolate this finding to a group of patients with severe glycemic control. However, the results from external validation on synthetic CGM data from people with different HbA1c levels could indicate that the model is generalizable.

In future perspectives, models such as the one proposed in this study need to be evaluated in a clinical impact study to assess the effects and clinical implications. The hypothesis is that the accurate prediction of hypoglycemic events could lead to better glycemic control with fewer events, increased time in range and less glycemic variability.

In conclusion, this work demonstrates the potential of using ensemble learning to predict hypoglycemia, using only CGM data, in a large and heterogeneous group of patients with type 1 diabetes.

## Data availability statement

The datasets presented in this study can be found online. The names of the links can be found below: the SCGMS database- 10.1177/19322968211014255; the REPLACE-BG database - https://diabetesjournals.org/care/article/40/4/538/3687/REPLACE-BG-A-Randomized-Trial-Comparing-Continuous; the original data of this study- https://public.jaeb.org/dataset/546.

## Ethics statement

The studies involving human participants were reviewed and approved as part of the original study REPLACE-BG listed on ClinicalTrials.gov under identifier NCT02258373. The patients/participants provided their written informed consent to participate in this study.

## Author contributions

SC had access to all the data analyzed in this study. SC takes responsibility for the integrity and accuracy of the study data analysis and results. SC and JF were involved in the study design, concept, analysis, and interpretation of the data. SC drafted the manuscript and performed the statistical analysis. JF and TH were involved in critical revision of the manuscript. All authors contributed to the article and approved the submitted version.
